# Supplementary material for: Making the effect visible – OX40 targeting nanobodies for in vivo imaging of activated T cells
Source: Front Immunol. 2024 Oct 15;15:1480091. doi: 10.3389/fimmu.2024.1480091 (PMC11518761; doi:10.3389/fimmu.2024.1480091)
Supplement: Supplementary file 1 [file DataSheet1.pdf]

Supplementary Information

Making the effect visible – OX40 targeting nanobodies for *in vivo* imaging of activated T cells

Desiree I. Frecot[1,2], Simone Blaess[3,4], Teresa R. Wagner[3], Philipp D. Kaiser[3], Bjoern Traenkle[3], Madeleine Fandrich[3], Meike Jakobi[3], Armin M. Scholz[5], Stefan Nueske[5], Nicole Schneiderhan-Marra[3], Cécile Gouttefangeas[2,6,7], Manfred Kneilling[2,4,8], Bernd J. Pichler[2,4,7], Dominik Sonanini[2,3,4,9], Ulrich Rothbauer[1,2#]

Affiliations

[1] Pharmaceutical Biotechnology, University of Tübingen, Tübingen, Germany

[2] Cluster of Excellence iFIT (EXC2180) "Image-Guided and Functionally Instructed Tumor Therapies", University of Tübingen, Tübingen, Germany

[3] NMI Natural and Medical Sciences Institute at the University of Tübingen, Reutlingen, Germany

[4] Werner Siemens Imaging Center, Department of Preclinical Imaging and Radiopharmacy, University of Tübingen, Tübingen, Germany

[5] Livestock Center of the Faculty of Veterinary Medicine, Ludwig Maximilians University Munich, Oberschleissheim, Germany

[6] Institute for Immunology, University of Tübingen, Tübingen, Germany

[7] German Cancer Consortium (DKTK) and German Cancer Research Center (DKFZ) partner site Tübingen, Tübingen, Germany

[8] Department of Dermatology, University of Tübingen, Tübingen, Germany

[9] Department of Medical Oncology and Pneumology, University of Tübingen, Tübingen, Germany

[#] corresponding author

Prof. Dr. Ulrich Rothbauer, Pharmaceutical Biotechnology, Pharmaceutical Institute, Auf der Morgenstelle 8, 72076 Tübingen, Germany

E-Mail: ulrich.rothbauer@uni-tuebingen.de

Phone: +49 7071 29 72469

Fax: +49 7071 29-2476

ORCID: 0000-0001-5923-8986

Supplementary Figures

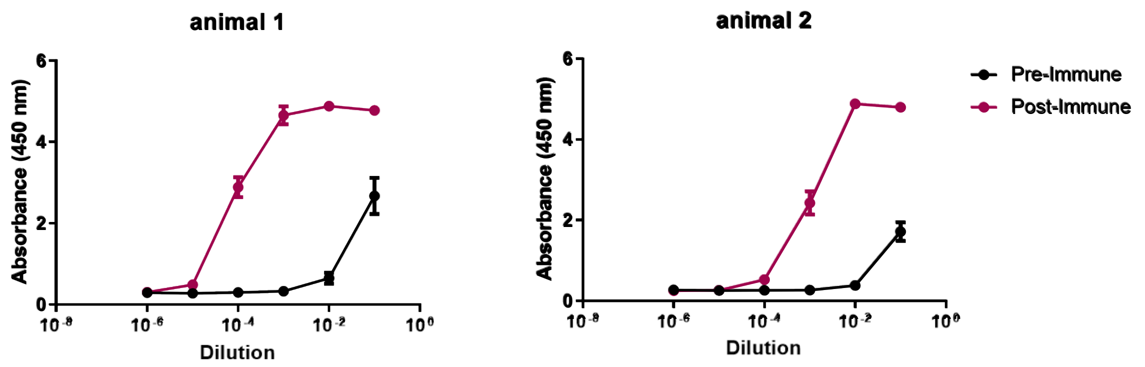

Fig. S1: Analysis of seroconversion upon vaccination with hOX40

Analysis of seroconversion upon vaccination with recombinant hOX40. Serum samples of the two vaccinated alpacas (*Vicugna pacos*) were collected before (pre-immune; black data points) and after 63 days of vaccination (post-immune; red data points). To test for the development of hOX40-specific antibodies, a serum ELISA was performed with the indicated dilutions of pre- and post-immune sera in multi-well plates coated with recombinant hOX40. Binding of hOX40-specific antibodies was detected by using an anti-heavy chain antibody conjugated to horseradish peroxidase.

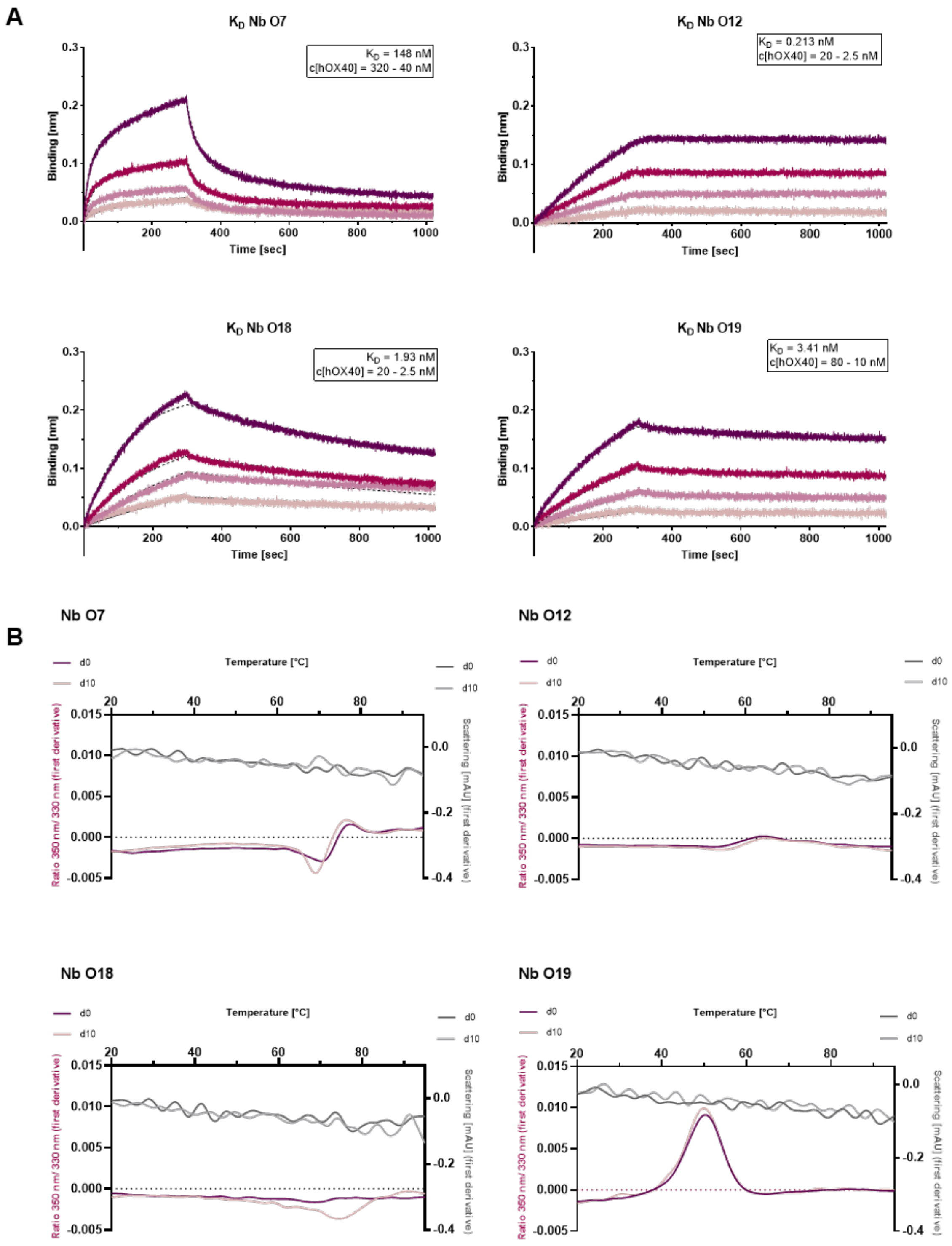

Fig. S2: Affinity and stability measurements of hOX40-Nbs

(**A**) Biolayer interferometry (BLI)-based affinity measurements were performed by immobilization of biotinylated hOX40-Nbs on streptavidin sensors. Kinetic measurements were performed using four concentrations (as indicated) of recombinant hOX40 (displayed with gradually lighter shades of color). The indicated binding affinities (K_D) were calculated from

global 1:1 fits shown as dashed lines. (**B**) Stability analysis of individual hOX40-Nbs using nano scale differential scanning fluorimetry (nanoDSF) displaying fluorescence ratio (350 nm/330 nm) (red) and light scattering (gray) are shown as first derivatives for day 0 (dark shade) and after an accelerated aging period of 10 days at 37°C (light shade).

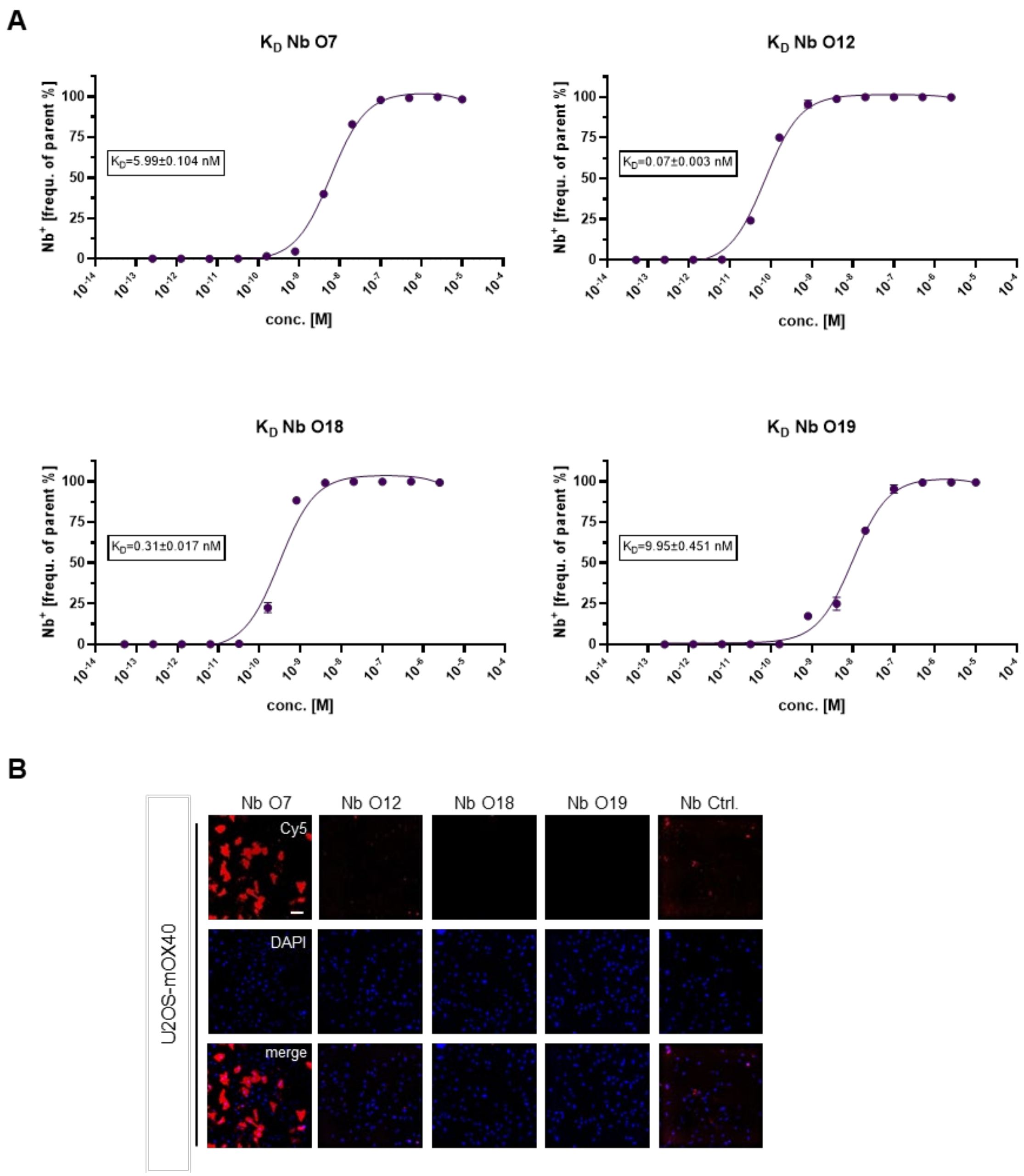

Fig. S3: Characterization of cellular binding of hOX40-Nbs

(**A**) Determination of hOX40-Nb binding to cellular expressed hOX40 by flow cytometry. The percentage of positively stained U2OS-hOX40 cells with AlexFluor647- (AF647) labeled hOX40-Nb (frequency of parent) was plotted against indicated concentrations of hOX40-Nbs and indicated K_D values were calculated from a four-parametric sigmoidal model based on the mean ± SD of three replicates (n = 3). (**B**) Cross-reactivity analysis of hOX40-Nbs by immunofluorescence staining. Representative images of U2OS cells transiently expressing murine OX40 (U2OS-mOX40) cells stained with 1000 nM AF647-labeled hOX40-Nbs or a non-

binding AF647-labeled PEP-Nb (Nb Ctrl.) as negative control. Shown are stainings with individual Nbs (red), nuclei staining (Hoechst, blue) and merged signals; scale bar: 50 µm.

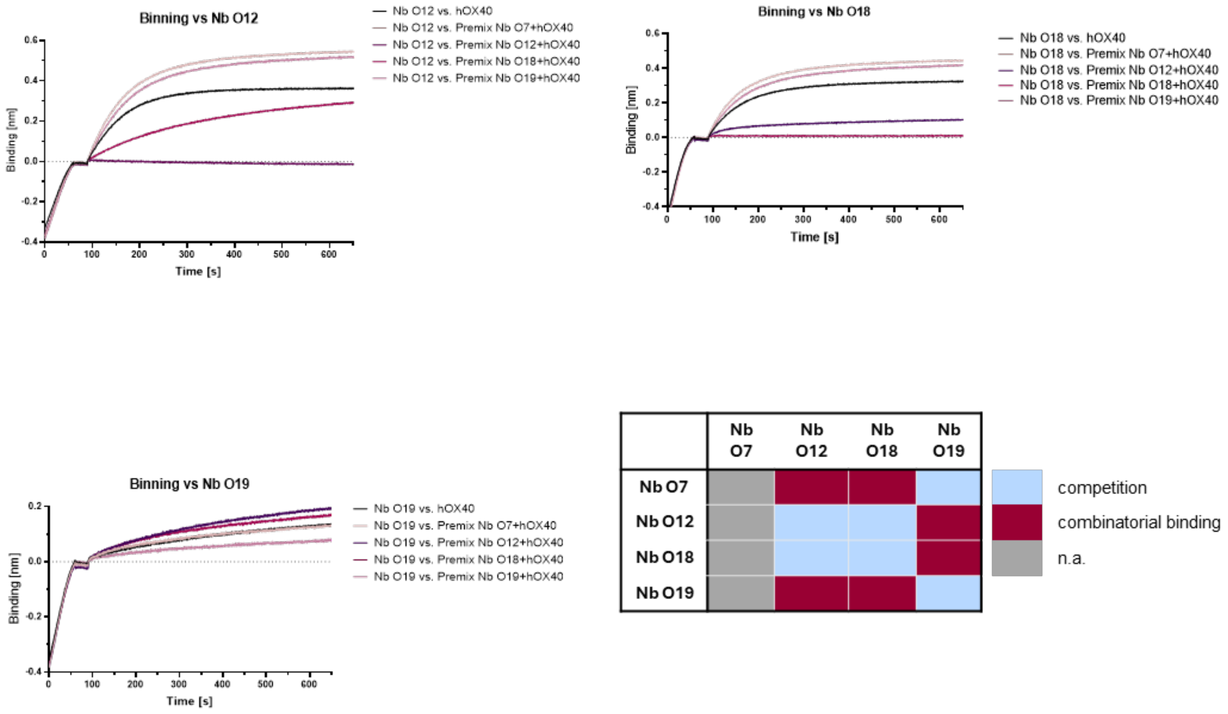

Fig. S4: Epitope binning of hOX40-Nbs by biolayer interferometry (BLI)

Biotinylated first hOX40-Nb was immobilized on streptavidin biosensors. hOX40 (100 nM) was pre-incubated (premix) in 10-fold excess with designated second hOX40-Nb (as indicated) followed by determining additional association of the hOX40/Nb complex to the immobilized first hOX40-Nb. All sensograms of combinatorial Nb binding to hOX40 on sharing/overlapping epitopes or on different epitopes including a graphical summary of epitope binning analysis are shown.

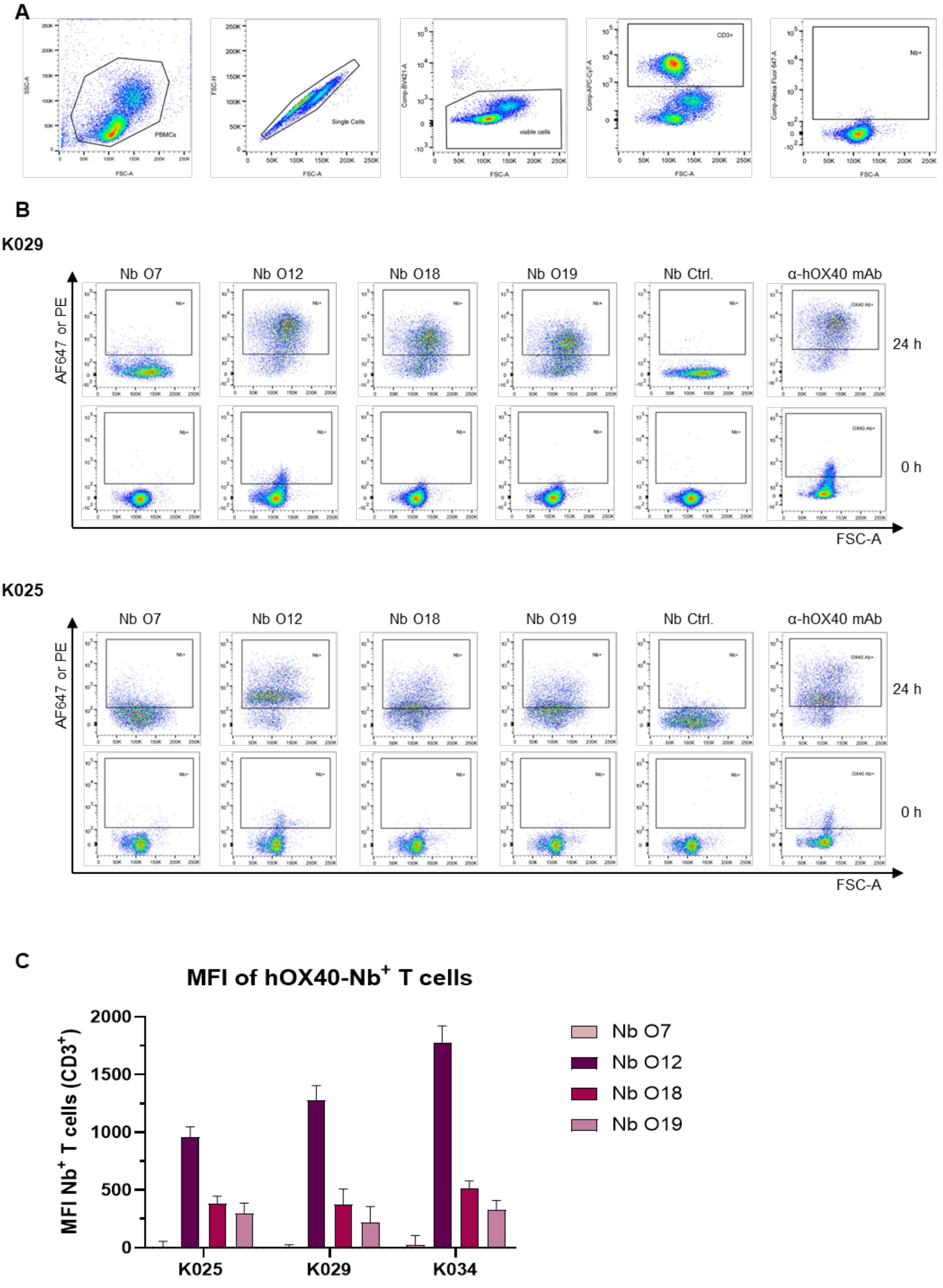

Fig. S5: Validation of hOX40-Nb binding to activated T cells

(**A**) Gating strategy for flow cytometry analysis of hOX40-Nb staining of CD3^{+} cells derived from human PBMCs (hPBMCs). Isotype control mAbs were used for setting the gates of anti-CD3 mAb and anti-hOX40 mAb (**B**) Flow cytometry analysis of CD3^{+} hPBMCs derived from

donors K029 and K025 stained with AF647-labeled hOX40-Nbs, a non-binding AF647-labeled PEP-Nb (Nb Ctrl.) or phycoerythrin (PE)-labeled anti-hOX40 mAb before (0 h, lower panel) and after (24 h, upper panel) stimulation with 5 µg/mL phytohaemagglutinin L (PHA-L) and 50 U/mL IL-2. (**C**) Mean fluorescence intensity (MFI) of hOX40-Nb positive T cells ($CD3^+$) from three different donors (K025, K029 and K034) after 24 h of PHA-L and IL-2 stimulation normalized to the signals of the Nb Ctrl. are shown as bar graph. Data are presented as mean ± SD of three replicates.

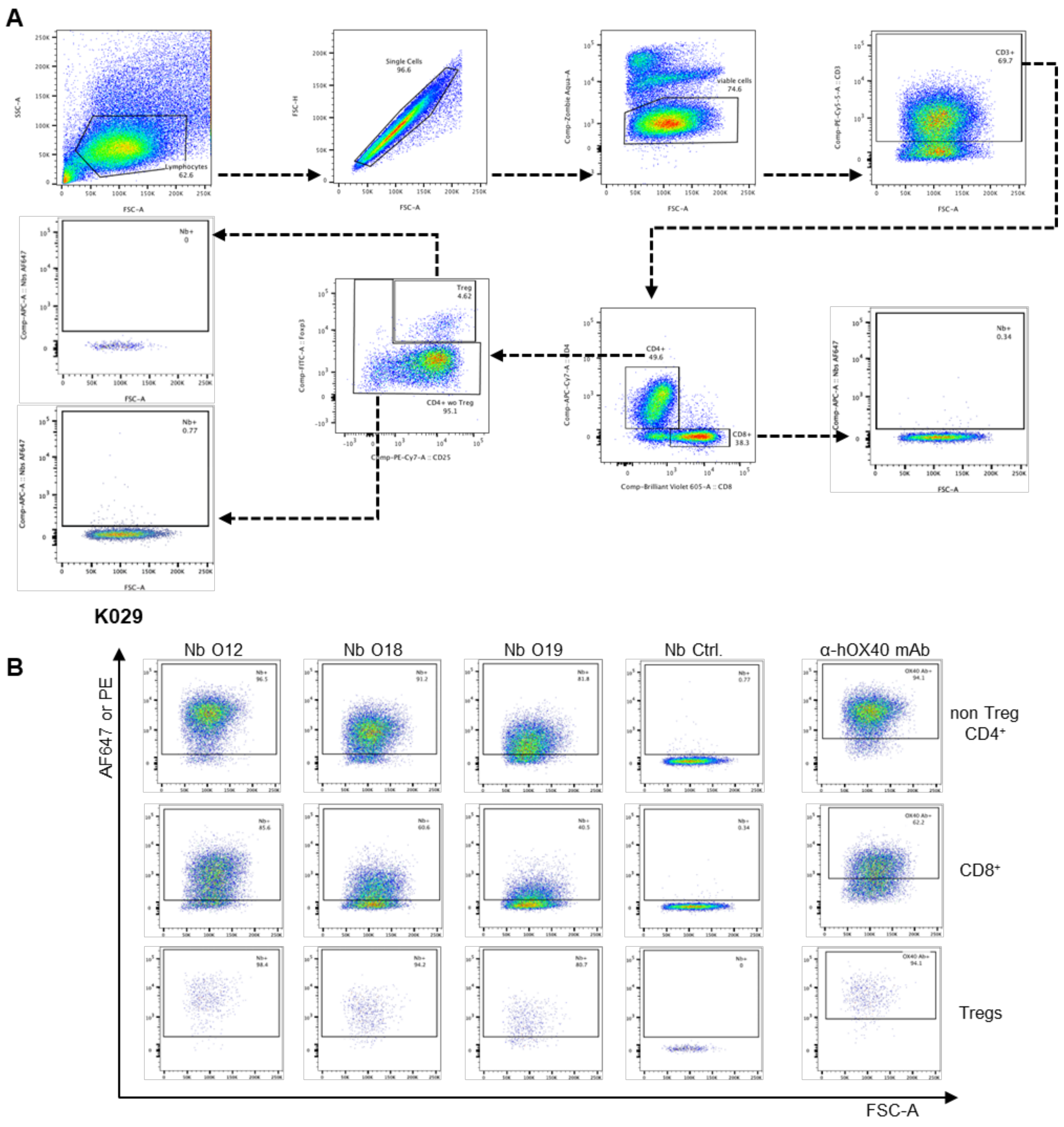

Fig. S6: Flow cytometry analysis of hOX40-Nb binding to activated T cell subpopulations

(**A**) Gating strategy for flow cytometry analysis of hOX40-Nb staining of non Treg CD4$^+$ T cells (CD3$^+$CD4$^+$FoxP3$^-$), CD8$^+$ T cells and regulatory T cells (Tregs, CD4$^+$CD25$^+$FoxP3$^+$) from human PBMCs (hPBMCs). Isotype control mAbs were used for setting the gates for anti-CD25 and anti-FoxP3 mAbs. (**B**) Exemplary analysis of non Treg CD4$^+$ T cells (upper panel), CD8$^+$ T cells (middle panel) and Tregs (lower panel) for donor K029 PBMCs stained with AF647-labeled hOX40-Nbs, a non-binding AF647-labeled PEP-Nb (Nb Ctrl.) or phycoerythrin (PE)-

labeled anti-hOX40 mAb after 24 h stimulation with 5 μg/mL phytohaemagglutinin L (PHA-L) and 50 U/mL IL-2.

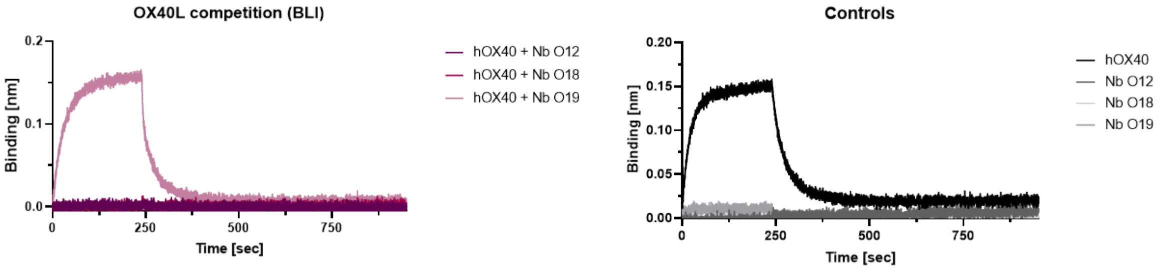

Fig. S7: Nb O12 and O18 compete with binding of OX40L to recombinant hOX40

BLI-based OX40L competition assay. OX40L was immobilized to the streptavidin sensors followed by addition of hOX40 either left untreated or preincubated with a 10-fold molar excess of Nb O12, O18 or O19. To exclude artefacts binding of each analyte to hOX40 was also tested alone (Controls).

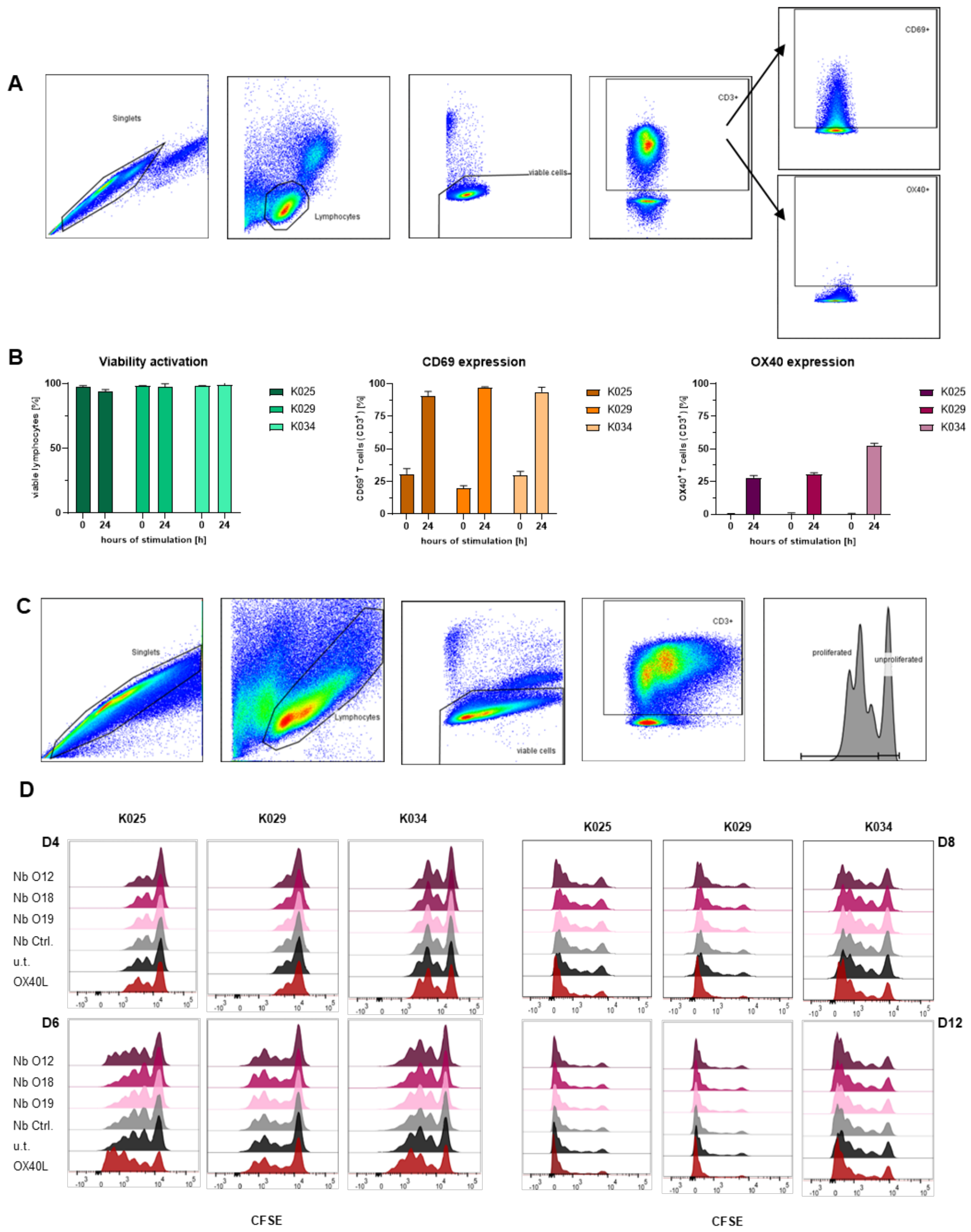

Fig. S8: Flow cytometry analysis of effects of hOX40-Nb binding

(**A**) Gating strategy for monitoring successful activation and hOX40 expression on hPBMCs upon PHA-L stimulation (exemplary shown for K034 at day 0). From left to right: single cells, lymphocytes, viable cells, CD3$^+$ cells and CD69 expressing (upper gate) or OX40 expressing (lower gate) cells. Isotype control mAbs were used for setting the gates of anti-CD3, anti-hOX40 and anti-CD69 mAbs. (**B**) Viability (left), CD69 expression (mid) and OX40 expression (right) before and after stimulation for the three donors K025, K029 and K034. Data are

presented as mean ± SD of three replicates (n = 3). (**C**) Gating strategy for T cell proliferation assay (exemplary shown for K034, 4 days after stimulation). From left to right: single cells, lymphocytes, viable cells, $CD3^+$ cells and CFSE-low/negative cells. Isotype control mAb was used for setting the gates of anti-CD3 mAb. (**D**) Histogram overlay shows the number of divisions as CFSE labeling within $CD3^+$ cells. Shown are all donors (K025, K029, K034) at days 4 (upper left panel), 6 (lower left panel), 8 (upper right panel) and 12 (lower right panel).

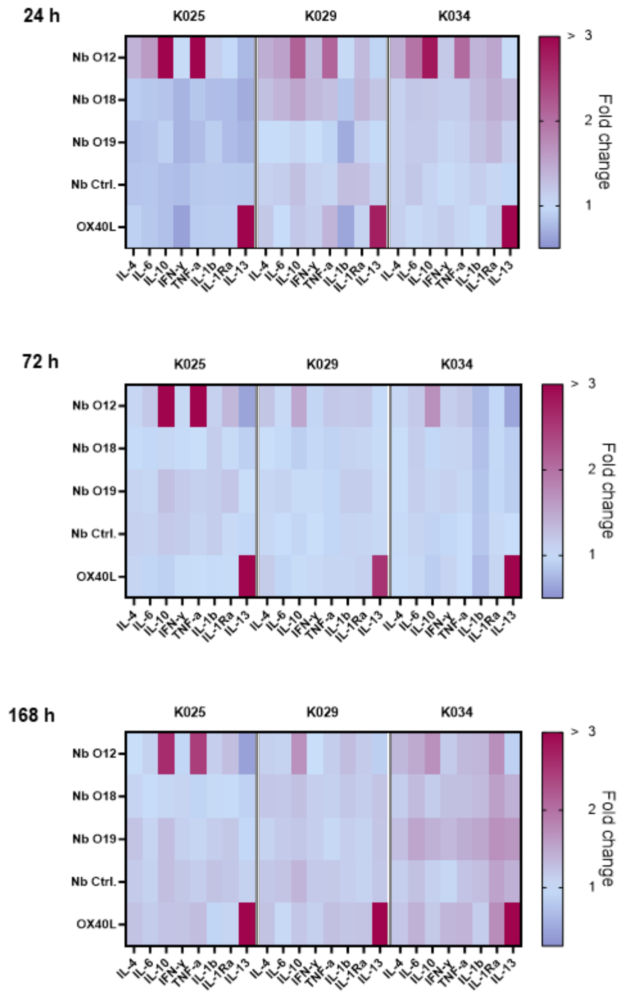

Fig. S9: Impact of hOX40-Nb treatment on cytokine release of hPBMCs

The impact of binding of hOX40-Nbs on the release of cytokine secretion was tested by treating hPBMCs of three donors (K25, K29, K34) with 0.5 µM hOX40-Nbs, non-binding PEP-Nb (Nb Ctrl.), OX40L or left untreated upon 24 h stimulation with 5 µg/mL PHA-L, to induce OX40 expression. Cytokine release to the medium was monitored at 24, 72 and 168 hours (24 h, 72 h, 168 h) after Nb treatment by a microsphere-based sandwich immunoassay (see **Supplementary Table S2**) and summarized in a heat map for each time point. Values are shown as fold change compared to the untreated control based on the mean of three technical replicates (n = 3).

Supplementary Tables

Table S1 Amino acid sequences and alignment of identified hOX40-Nbs

hOX40-Nb	Amino acid sequence
**O7**	DVQLVESGGGLVQPGGSLRLSCAASGFTFGSYAMGWARQVPGKGLEWVSGIYSDGSTYYADSVKGRFTI SRDNAKNTVYLQMNSLKPEDTAVYYCATWGAAAPYDYWGQGTQVTVSS
**O12**	DVQLQESGGGLVQPGGSLRLSCAASGFTLDNYAIGWFRQAPGKEREGVSCISSSGESTNYADSVKGRFTI SRDNAKNTVYLQTNSLKPEDTAVYYCAAVDDIGTTVQFMCNMGPYEYDYWGQGSPVTVSS
**O18**	QVQLVESGGGLVQPGGSLRLSCAASGFTLEDYVIGWFRQAPGKEREEVSCISGSGGIRNVADSMEGRATI SRDNAKNTVYLQMNSLKPEDTAVYYCAAGFETSYSYSYYCGVHEYDYWGQGTLVTVSS
**O19**	QVQLQESGGGLVQPGGSLRLSCAGSTDTFSMSAAVSWARQAPGKGLEWVSSILSDGSTYYADSVRGRF TISRDNANNTGSLQMNNLKVEDTAVYYCTNLRGRLWSNHKDDYGGQGSRVTVSS

```
     -------Framework 1-----  --CDR1---  ----Framework2----  ----CDR2----  -------------Framework3-------------  --------CDR3--------  -Framework4-
O7  DVQLVESGGGLVQPGGSLRLSCAA--SGFTFG-SY--AMGWARQVPGKGLEWVSG--IYSD-GSTYYAD--SVKGRFTISRDNAKNTVYLQMNSLKPEDTAVYYCAT--WG------------AAAPYD--YWGQGTQVTVSS

O12 DVQLQESGGGLVQPGGSLRLSCAA--SGFTLD-NY--AIGWFRQAPGKEREGVSC--ISSSGESTNYAD--SVKGRFTISRDNAKNTVYLQTNSLKPEDTAVYYCAA--VDDIGTTVQFMCNMGPYEYD--YWGQGSPVTVSS

O18 QVQLVESGGGLVQPGGSLRLSCAA--SGFTLE-DY--VIGWFRQAPGKEREEVSC--ISGSGGIRNVAD--SMEGRATISRDNAKNTVYLQMNSLKPEDTAVYYCAA--GFETSYSYSYYC--GVHEYD--YWGQGTLVTVSS

O19 QVQLQESGGGLVQPGGSLRLSCAG--STDTFSMSA--AVSWARQAPGKGLEWVSS--ILSD-GSTYYAD--SVRGRFTISRDNANNTGSLQMNNLKVEDTAVYYCTN--LRGRLW--------SNHKDD--YGGQGSRVTVSS

    :*** ******************.  *  *:  .   .:.* **.***  * **   * ..      **  *:.** *******:**  ** *.** ********:                 .    *  * ***: *****
```

Table S2 Cytokines analyzed in this study

Cytokine	indicative for
Interleukin 1b (IL-1b)	proinflammatory
Interleukin 1 receptor antagonist (IL-1Ra)	antiinflammatory
Interleukin 4 (IL-4)	antiinflammatory
Interleukin 6 (IL-6)	pro-/antiinflammatory
Interleukin 10 (IL-10)	antiinflammatory
Interleukin 13 (IL-13)	antiinflammatory
Interferon γ (IFN-γ)	proinflammatory
Tumor necrosis factor α (TNF-α)	proinflammatory

Table S3 primers used in this study

Name	Sequence 5'- 3'	purpose
CALL001	GTCCTGGCTGCTCTTCTACAAGG	Nb library generation
CALL002	GGTACGTGCTGTTGAACTGTTCC	
FR1-1	CATGGCNSANGTGCAGCTGGTGGANTCNGGNGG	
FR1-2	CATGGCNSANGTGCAGCTGCAGGANTCNGGNGG	
FR1-3	CATGGCNSANGTGCAGCTGGTGGANAGYGGNGG	
FR1-4	CATGGCNSANGTGCAGCTGCAGGANAGYGGNGG	
FR1-ext1	GTAGGCCCAGCCGGCCATGGCNSANGTGCAGCTGGTGG	
FR1-ext2	GTAGGCCCAGCCGGCCATGGCNSANGTGCAGCTGCAGGA	
FR4-1	GATGCGGCCGCNGANGANACGGTGACCNGNRYNCC	
FR4-2	GATGCGGCCGCNGANGANACGGTGACCNGNGANCC	
FR4-3	GATGCGGCCGCNGANGANACGGTGACCNGRCTNCC	
FR4-4	GATGCGGCCGCRCTNGANACGGTGACCNGNRYNCC	
FR4-5	GATGCGGCCGCRCTNGANACGGTGACCNGNGANCC	
FR4-6	GATGCGGCCGCRCTNGANACGGTGACCNGRCTNCC	
Ox40_2-4_1for	CAGTACATCAATGGGCGTGG	hOX40 domain deletions
Ox40_2-4_1rev	GAGAGACTGCACGCACGCGATCCGGCATGGTGGCGCTAGCCAG	
Ox40_2-4_2for	CGTGCGTGCAGTCTCTCACTGGAGCAGCCCGTGCGGGCCGG	
Ox40_2-4_2rev	GCGGAATTCTCAGATCTTGGC	
Ox402-4_3for	GGGAGACCCAAGCTGGCT	
Ox40_P1-rev	GGCTGCTCCAGTGAGAGACTGCACGCACGC	
Ox40_3-4-for	GCAGTCTCTCACTGGAGCAGCCGATGTCGAGCTGGCACCCAGCCCCTG	
Ox40_4-for	GCAGTCTCTCACTGGAGCAGCCCTTGTCCTCCAGGGCACTTCTCCC	
